# Deep brain stimulation restores the glutamatergic and GABAergic synaptic transmission and plasticity to normal levels in kindled rats

**DOI:** 10.1371/journal.pone.0224834

**Published:** 2019-11-07

**Authors:** Samireh Ghafouri, Yaghoub Fathollahi, Saeed Semnanian, Amir Shojaei, Azam Asgari, Azin Ebrahim Amini, Javad Mirnajafi-Zadeh

**Affiliations:** 1 Department of Physiology, Faculty of Medical Sciences, Tarbiat Modares University, Tehran, Iran; 2 Département de Neurosciences, Université de Montréal, Montréal, Canada; 3 Department of Biomaterial and Biomedical Engineering (IBBME), Faculty of applied sciences, University of Toronto, Toronto, Canada; 4 Institute for Brain Sciences and Cognition, Tarbiat Modares University, Tehran, Iran; University of Modena and Reggio Emilia, ITALY

## Abstract

**Background:**

The precise effect of low frequency stimulation (LFS) as a newly postulated, anticonvulsant therapeutic approach on seizure-induced changes in synaptic transmission has not been completely determined.

**Hypothesis:**

In this study, the LFS effect on impaired, synaptic plasticity in kindled rats was investigated.

**Methods:**

Hippocampal kindled rats received LFS (4 trials consisting of one train of 200 monophasic square waves, 0.1 ms pulse duration, 1 Hz) on four occasions. LTP induction was evaluated using whole-cell recordings of evoked excitatory and inhibitory post-synaptic potentials (EPSPs and IPSPs respectively) in CA1 neurons in hippocampal slices. In addition, the hippocampal excitatory and inhibitory post-synaptic currents (EPSCs and IPSCs), and the gene expression of NR_2_A, GluR_2_ and γ_2_ were evaluated.

**Results:**

LTP induction was attenuated in excitatory and inhibitory synapses in hippocampal slices of kindled rats. When LFS was applied in kindled animals, LTP was induced in EPSPs and IPSPs. Moreover, LFS increased and decreased the threshold intensities of EPSCs and IPSCs respectively. In kindled animals, NR_2_A gene expression increased, while γ_2_ gene expression decreased. GluR2 gene expression did not significantly change. Applying LFS in kindled animals mitigated these changes: No significant differences were observed in NR_2_A, γ_2_ and GluR_2_ gene expression in the kindled+LFS and control groups.

**Conclusion:**

The application of LFS in kindled animals restored LTP induction in both EPSPs and IPSPs, and returned the threshold intensity for induction of EPSCs, IPSCs and gene expression to similar levels as controls.

## Introduction

Synaptic plasticity is the most common physiological phenomenon in learning and memory. It is the sum of the changes in synaptic strength, which potentiate or depress over time in response to increments or decrements in synaptic activity [1]. In addition to excitatory synapses, various types of inhibitory plasticity have been also reported; these depend on the inhibitory interneurons, cell type and brain region [2]. Inhibitory plasticity occurs as a result of changes in either presynaptic GABA release or the number, sensitivity, or responsiveness of postsynaptic GABA_A_ receptors [3]. This type of synaptic plasticity plays a pivotal role in neuronal circuit refinement by altering the excitatory/inhibitory balance [4].

In some pathological conditions, such as epilepsy, disruption in synaptic plasticity results in learning and memory impairment [5]. In many epileptic patients, the epileptogenic region is located within the mesial temporal structures, including amygdala, parahippocampal gyrus, and hippocampus [6]. Since the hippocampus is a common, epileptogenic brain structure, it has been widely used for kindling models [7].

Electrical kindling is an experimental model for complex partial seizures with secondary generalization, and electrical kindling involves the application of repeated, sub-threshold electrical stimulation to specific brain regions, leading to a permanent increase in seizure susceptibility [8]. The kindling procedure is accompanied by a special kind of synaptic potentiation that is highly similar to excitatory long-term potentiation (eLTP) [9, 10]. In addition, previous experiments [11, 12] showed that semi-rapid kindling (in which the animals receive 10–12 stimulations per day) can induce synaptic potentiation, which is similar to potentiation induced during traditional kindling (one stimulation per day) [11, 12]. This potentiation reduces the ability of the hippocampal synapses to express new plasticity [13].

The anticonvulsant effect of different patterns of deep brain stimulation (DBS), such as low frequency stimulation (LFS), has been reported in epileptic patients and kindled animals [14, 15]. In fact, DBS is FDA approved for treatment of epilepsy [16, 17]. However, its total impact on brain function is yet to be elucidated.

LFS has profound and long-lasting effects on seizure development, expression, and thresholds [18]. Our lab has previously demonstrated the antiepileptic effect of LFS. Furthermore, LFS may also prevent kindling-induced potentiation and spatial working memory impairments [11, 12, 19]. However, the application of LFS can induce long-term depression (LTD) and depotentiation [20, 21]. It has been suggested that the LFS anticonvulsant mechanisms may be similar to mechanisms involved in LTD or depotentiation. Thus, in the present study, we investigated whether applying LFS as a potential anticonvulsant, therapeutic strategy can prevent the impairment of LTP induction in excitatory and inhibitory synapses in the hippocampal CA1 region.

## Materials and methods

### Animal surgery

Five-to-six week old male Wistar rats were housed individually in cages with an ambient temperature of 22°C—25°C, 12-h light/12-h dark cycle (lights on from 7:00 a.m. -7 p.m.), and provided with water and food ad libitum. All experiments and animal handling were done according to international guidelines on the use of laboratory animals and approved by “Tarbiat Modares University Ethical Committee for Animal Research,” which is in line with the ‘‘NIH Guide for the Care and Use of Laboratory Animals.” Efforts were made to reduce the number of animals used and decrease their suffering.

Before surgery, the animals were anesthetized with a mixture of ketamine (100 mg/kg, i.p.) and xylazine (10 mg/kg, i.p.). The anesthetized rats were fixed in a stereotaxic frame and their skin was retracted to expose the skull. A tripolar electrode, consisting of a bipolar stimulating and a monopolar recording electrode (twisted together), was implanted in the CA1 region of the right hippocampus at 2.4 mm posterior and 1.8 mm lateral to the bregma, and 2.8 mm below the skull [22]. Electrodes were stainless steel, teflon-coated, and insulated except at their tips (A-M Systems, Inc., WA, U.S.A.; 127 μm in diameter). A monopolar reference/ground electrode, connected to a stainless steel screw, was positioned in the skull above the occipital cortex.

The electrode location was histologically determined in the implanted rats after their experiments were complete. Rats were anesthetized with isoflurane and perfused with 4% paraformaldehyde in 0.1 M phosphate buffer (pH 7.4). Then, their brains were extracted and sectioned to verify electrode placement. Only subjects with correct electrode placement were considered for data analysis.

### Hippocampal kindling

The kindling procedures were completed as previously reported [12]. After a post-surgical recovery period of 7 days, the afterdischarge (AD) threshold was determined by 1 ms monophasic square wave of 50 Hz with a 3 s train duration. The stimulating currents were initially delivered at 30 μA, then intensity was increased in increments of 10 μA at 10 min intervals until ADs of at least 8 s were recorded. ADs were defined as spikes with a frequency of at least 1 Hz and amplitude of at least twice the baseline activity originating immediately post stimulation. Rats were electrically stimulated at the AD threshold 12 times a day with an interval of 10 min. Epileptiform ADs were continuously recorded from the hippocampal CA1 area following kindling stimulations using a PC-based data acquisition system (D3107; ScienceBeam Co., Tehran, Iran).

Seizure severity ratings were based on the Racine scale [23]: Stage 0, rats showed no convulsion; Stage 1, rats showed facial automatism; Stage 2, head nodding; Stage 3, unilateral forelimb clonus; Stage 4, bilateral forelimb clonus; and Stage 5, rearing, falling and generalized convulsions. The animals were considered fully kindled when they exhibited stage 5 on three consecutive days.

### LFS application

LFS was applied 4 times at 30 seconds, 6, 18 and 24 hours following the last kindling stimulation. Each LFS consisted of 4 packages at 5 minutes intervals. Each package contained 200 monophasic square wave pulses of 0.1 ms duration at 1 Hz. The intensity of LFS was equal to AD threshold of each animal. The LFS pattern was achieved according to our preliminary experiments.

### Whole-cell patch clamp recording

Twenty-four hours after the last kindling stimulation (2–3 hours following the last LFS application), electrophysiological experiments were conducted. Hippocampal slices were prepared in vitro as follows. Rats were anesthetized with isoflurane and decapitated. Brains were rapidly extracted, and the right hemispheres were dissected. Tissue was placed in ice-cold cutting solution containing (in mM) 2.5 KCl, 0.5 CaCl_2_, 2 MgCl_2_, 1 NaH_2_PO_4_, 26.2 NaHCO_3_, 238 sucrose and 11 D-glucose and bubbled with 95% O2- 5% CO2. Osmolarity was adjusted to 290–300 mOsm, and the pH was adjusted to 7.2–7.35 in the cutting solution. Transverse slices (400 μm) were cut using a vibratome (1000 Plus Sectioning System, Vibratome, MO, USA). Subsequently, the sliced right hippocampi were incubated for 1 hour at 32°C. For incubation, we used standard ACSF that was continuously bubbled with 95% O_2_- 5% CO_2_, and contained (in mM): 125 NaCl, 3 KCl, 1.25 NaH_2_PO_4_, 25 NaHCO_3_, 10 D-Glucose, 2 CaCl_2_, 1.3 MgCl_2_. Osmolality was in the range of 290–300 mOsm and pH was adjusted to 7.2–7.35 by NaOH 1 M. Then slices were individually transferred to a submerged recording chamber.

The recording chamber was mounted on a fixed-stage, upright microscope (Axioskop 2 FS MOT; Carl Zeiss, Germany) and was continuously perfused at 1.5–2 ml/minutes with standard ACSF at room temperature (24 ± 1°C). CA1 pyramidal neurons were visualized using an IR-CCD camera (IR-1000, MTI, USA) with a 40× water immersion objective lens. Neurons were selected for recording based on their relative pyramidal shape and smooth, low-contrast appearance. Whole-cell patch clamp recordings were made under voltage clamp mode. Recording microelectrodes (1.5 mm outer diameter, borosilicate glass, GC150-11; Harvard Apparatus, UK) were pulled with a horizontal puller (P-97, Sutter Instrument, USA).

For excitatory LTP (eLTP) induction in excitatory synapses and EPSC recording, glass microelectrodes were filled with intracellular solution containing (in mM): 135 K-gluconate, 20 KCl, 10 HEPES, 0.2 EGTA, 7 disodium-phosphocreatine, 2 MgATP, 0.3 NaGTP and 1 QX-314. For inhibitory LTP induction (iLTP) in inhibitory synapses and eIPSC recording, the intracellular solution contained (in mM): 140 CsCl, 1 CaCl2, 5 QX-314, 10 HEPES, 2 MgCl2, 2 Mg-ATP, 2Na-GTP and 0.5 EGTA. pH was set at 7.2–7.35 and osmolality was in the range of 290–300 mOsm. The electrode tip resistance in the bath was 5 to 7 MΩ, and series resistance ranged from 18 to 30 MΩ. Cells were rejected if the series resistance was changed more than 20% during the experiment. Capacitance compensation and bridge balance were carried out. Data were low-pass filtered at 3 kHz and acquired at 10 kHz with a Multiclamp 700B amplifier equipped with Digidata 1440 A/D converter (Molecular Devices, CA, USA). The signal was recorded on a PC for offline analysis using the Axon pClamp 10 acquisition software. After establishment of a gigaseal (more than 2 GΩ), the whole-cell configuration was achieved by applying a brief suction.

Synaptic responses were evoked by constant monophasic current pulses (0.1 ms, 50–200 μA) delivered through a stimulus isolation unit (WPI-A360, World Precision Instruments, Sarasota, FL, USA) to a stimulating electrode (bipolar teflon-coated, stainless steel electrodes; A-M Systems, Inc., WA, U.S.A.; 127 μm in diameter); placed in the Schaffer collateral pathway to record excitatory monosynaptic responses and placed in the striatum radiatum to record inhibitory monosynaptic responses. The distance between stimulating and recording electrodes was less than 50 μm for inhibitory synapses and 100–150 μm for excitatory synapses. Minimal stimulation protocol was used to determine the threshold intensity and 1.5×threshold intensity to record excitatory and inhibitory monosynaptic responses respectively.

Monosynaptic excitatory responses were recorded following the addition of bicuculline (GABA_A_ receptor antagonist, 20 μM; Tocris Bionscience, England) into ACSF. Monosynaptic inhibitory responses were pharmacologically isolated using 6-cyano-7-nitroquinoxaline-2,3-dione (CNQX; AMPA receptor antagonist, 20 μM; Tocris Bionscience, England) and (±)-2-amino-5-phosphopentanoic acid (AP5;NMDA receptor antagonist, 50 μM; Tocris Bionscience, England) to block non-NMDA and NMDA receptors, respectively.

High frequency stimulation (HFS) was used for LTP induction both in excitatory synapses (2 trains of 100 pulses at 100 Hz for 1 s, 20-s interval) and inhibitory synapses (2 trains of 7 pulses at 100 Hz for 1 s, 20-s interval).

### RNA extraction, cDNA synthesis and quantitative real time-PCR experiments

For quantitative, real time-PCR (RT-qPCR), animals were anesthetized with CO2, sacrificed, and their hippocampi were isolated 24–27 hours after the last kindling stimulation, and preserved at −80°C temperature, based on our previous experiments [19].

(a) *RNA preparation and reverse transcription*: For gene expression study, total mRNA was isolated based on the phenol-chloroform extraction method [24], using total extraction kit (Parstous, Iran) according to the manufacturer’s instructions. The final total RNA pellet was air-dried, and the RNA was suspended in 30 μl of DEPC (diethyl-pyrocarbonate)-treated water. One μl of total RNA was used for spectrophotometric determination of the RNA concentration at 260 and 280 nm. Two μl of total RNA were used to determine integrity and quality of RNA samples extracted from electrophoresis (Akhtarian, Iran) on 1% agarose gel (Fermentaz, Germany). The extracted RNA was entered into the cDNA construction phase. For each sample, cDNA synthesis was performed using 2 μg of total RNA, 2 μL Oligo-dT primer (Parstous, Iran), and 10 μL reverse transcriptase (Parstous, Iran) according to the manufacturer’s instructions.

(b) *RT-qPCR*: The cDNA pool was subjected to RT-qPCR by using a Real Q-PCR Master Mix Kit (Ampliqon, Herlev, Denmark) on a Rotor-Gene Q device (Qiagen, Hilden, Germany). The following conditions were used for RT-qPCR: Initial heating for 15 minutes at 95°C; 35 cycles of amplification, each composed of 60 s at 95°C; 60 s at the annealing temperature; and 60 s at 72°C. The annealing temperature for NR_2_A, GluR_2_, γ_2_ and GAPDH was 60°C. GAPDH was used as an endogenous control to minimize the effect of sample variations in calculating the relative expression level of target genes by the delta–delta-Ct method. Cycle threshold was measured as an index of gene expression. In our experiments, there was no significant difference in cycle threshold for GAPDH in different experimental groups; therefore, we considered it a suitable housekeeping gene. Primer sequences for NR2A, GluR2, γ2 and GAPDH were purchased from CinnaGen, Iran (Table 1).

**Table 1:** Sequences of primers used in real-time polymerase chain reactions.

Gene	Primer sequence	Product length	Accession number
PGK 1	F:GCCAAGTCGGTTGTGCTTATG R: CCAGGAGGATGACAGTCCCA	196	NM_053291.3
GAPDH	F: TCCCATTCTTCCACCTTTGATGCT R:ACCCTGTTGCTGTAGCCATATTCAT	104	NM-017008
Calcineurin A-α	F:TGACCACTTCCTGTTCACTTTTTTT R: GCAAGAACATCCAACTGCTGAG	80	NM-017041.1
gama2 GABAA	F: ACCATAGCCCGGAAGTCTCT R: CCTTGCTTGGTTTCCGGTTG	145	NM-183327.1
NR2A NMDA	F: CAATCTGACTGGATCACAGAGC R: CTGTCCTTCCCTTGAAAGGATC	207	NM-012573
GluR2 AMPA	F: ATGGTTCAGTTTTCCACTTCGG R: TGCGTAGACTCCTCTTGAAAACTG	120	NM-017261.2

### Experimental design

Animals were assigned to five groups: Kindled, kindled+LFS, control+LFS, sham, and control. In the kindled group, slice preparation and hippocampal sampling were completed 24 hours following the last kindling stimulation. In the kindled+LFS group, slice preparation and hippocampal sampling were performed 2–3 hours after LFS application. In the control+LFS group, animals were manipulated similar to kindled+LFS group, but received only LFS without kindling stimulations. In the sham group, animals underwent surgery but did not receive any stimulation. In this group, the elapsed time between the surgery, electrophysiological experiments, and hippocampal sampling were similar to animals of the kindled and kindled+LFS groups (about 15 days). In the control group, electrophysiological experiments and hippocampal sampling were conducted on intact animals.

### Statistical analysis

Data were averaged and expressed as mean ± S.E.M. Statistical analysis was performed using GraphPad Prism version 6.01 for Windows (GraphPad Software, Ca, USA). To check the normality of distribution of data, Kolmogorov-Smirnov test was used and the p-values were calculated for all experimental groups. Obtained results showed the normal distribution. To evaluate the effect of kindling and LFS application on amplitude of EPSP, EPSC, IPSP, IPSC, paired-pulse stimulation analysis, and NR_2_A, GluR_2_, γ_2_ gene expression in different groups, a two-way ANOVA with post-hoc Tukey’s tests were used. A p-value of less than 0.05 was considered a significant difference.

## Results

There was no significant difference in AD threshold (82.73 ± 10.37 μA in the kindled and 71.75 ± 7.73 μA in the kindled+LFS groups) and AD duration after the first kindling stimulation (23.85 ± 1.41 s in the kindled and 22.88 ± 1.60 s in the kindled+LFS groups) among different experimental groups. In addition, the mean number of stimulation days to achieve the fully kindled state was similar in the kindled (9.00 ± 0.39 days, n = 17) and kindled+LFS (8.18 ± 0.36 days, n = 16) groups. Therefore, seizure susceptibility was not different among experimental groups at the beginning of the experiments. There was no significant difference between the sham and control groups in all experiments, thus, their data were merged and considered as control.

**Fig 1:**
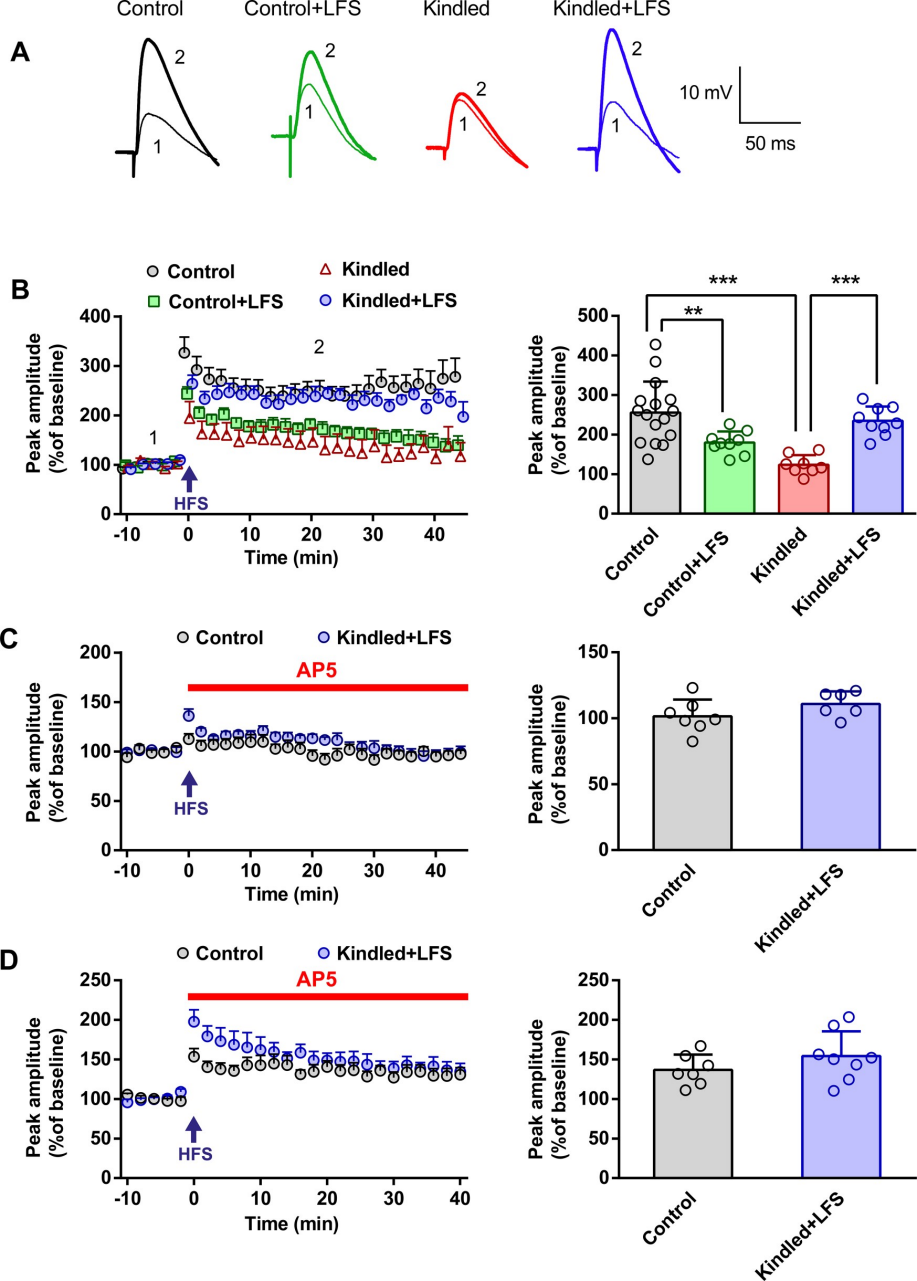
Improvement of eLTP induction in hippocampal slices of kindled animals following LFS application. (A) EPSPs sample recordings of different experimental groups, before (1) and after (2) HFS application. The numerals near the traces correspond to the times indicated by the same numerals in the graphs. (B) Left: Time course of the EPSP amplitude alteration (% of the baseline) within 40 min after HFS delivery. In the kindled group, eLTP was attenuated significantly compared to the control group. Also, impairment of eLTP induction was restored following LFS administration in the kindled animals. In addition, delivery of LFS alone in control animals decreased the magnitude of eLTP induction compared to the control group. Right: Bar graph shows the quantitative analysis of eLTP. Control: n = 16, Control+LFS: n = 9, Kindled: n = 8, Kindled+LFS: n = 9. (C) Investigating the role of NMDA receptors in eLTP induction in the control and kindled+LFS groups. Left: Time course and Right: bar graph represent that in the presence of AP5, HFS could not induce eLTP in the control or kindled+LFS group. Control: n = 7, Kindled+LFS: n = 6. (D) Investigation the role of NMDA receptors in maintaining eLTP in the control and kindled+LFS groups. Left: Time course and Right: bar graph show that eLTP was induced both in the control and kindled+LFS groups when AP5 was administered immediately after HFS application. Control: n = 7, Kindled+LFS: n = 8. Data are shown as mean±SEM. ** p<0.01 and *** p<0.001 compared to control group.

In the first experiment, we examined the effect of applying LFS on seizure-induced impairments in eLTP induction in kindled animals. For this purpose, EPSPs were recorded in current clamp mode, where the current was injected to maintain the membrane potential at -80 mV to prevent the spike generation after HFS application, for 10 minutes as a baseline. HFS protocol was applied in voltage clamp mode (V = -10 mV) using a stimulating electrode in the Schaffer collateral pathway. Then EPSPs were rerecorded in current clamp mode for 40 minutes. Data were shown as a percentage of the baseline. Following application of HFS, EPSP amplitude in the control group was 255.80 ± 19.54% of the baseline (n = 16); while in the kindled group, the amplitude of EPSPs was 123.90 ± 8.62% of the baseline (n = 8). A two-way ANOVA showed a significant difference between these two groups (P<0.001). The impairment of eLTP induction in kindled animals was restored following administration of LFS, so that there was no significant difference in HFS-induced potentiation in EPSP amplitude between the kindled+LFS (234.70 ± 12.06% of baseline, n = 9) and control groups. Interestingly, delivery of LFS alone in control animals decreased the magnitude of eLTP induction. There was a significant difference in potentiation of EPSP amplitude between the LFS (179.70±9.47% of baseline, n = 9) and control groups (P<0.01) (Fig 1B).

In the next step, we checked if the induction and maintenance of the observed eLTP in the kindled+LFS group is NMDA receptor dependent. Our results showed that applying HFS, in the presence of AP5, did not induce eLTP in the kindled+LFS (110.80 ± 3.87% of baseline, n = 6) or in the control (101.50±4.80% of baseline, n = 7) group (Fig 1C). However, when AP5 was applied immediately after HFS, eLTP was induced in the kindled+LFS (154.20 ± 11.09% of baseline, n = 8) and in the control (136.70 ± 7.34% of baseline, n = 7) group (Fig 1D). However, in the presence of AP5, compared to the absence of AP5 (control: 136.80±7.34% vs. 255.80±19.51% of baseline, P<0.001 and kindled+LFS: 154.20±11.09% vs. 234.70±12.06% of baseline, P<0.05), there was a significant reduction in the amplitude of EPSPs after HFS application both in the control and kindled+LFS groups.

**Fig 2:**
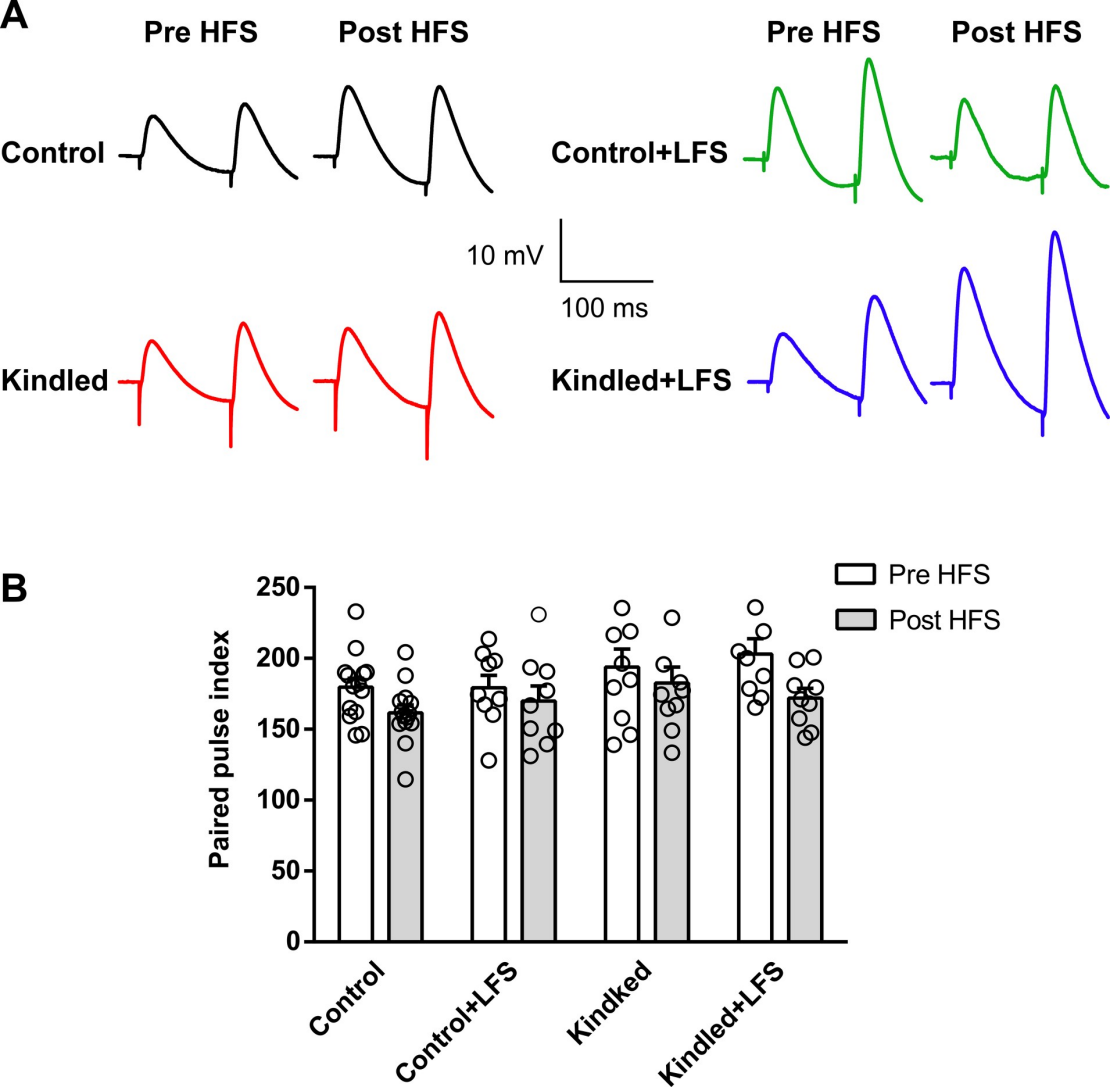
Effect of LFS on PPI of EPSPs before and after HFS application in hippocampal slices of different experimental groups. (A) Sample recording of EPSPs paired pulses in different experimental groups. No significant change in PPI of EPSPs was observed before and after HFS application in the control, control+LFS, kindled or kindled+LFS group. Control: n = 14, Control+LFS: n = 9, Kindled: n = 10, Kindled+LFS: n = 9. Data are shown as mean±SEM.

Paired pulse index (PPI) was also determined before and after HFS application. PPI was calculated as (the amplitude of the second EPSP divided by the amplitude of the first EPSP)×100. A two-way ANOVA revealed that there was no significant difference in PPI of different experimental groups before HFS delivery. Moreover, no significant change in PPI was observed before and after HFS application in the kindled (193.80 ± 12.70% vs. 182.60 ± 11.25%, n = 10), kindled+LFS (203.10 ±10.75% vs. 172.10 ± 6.75%, n = 9), LFS (179.20 ± 8.80% vs. 170.00 ± 10.55%, n = 9) and control (179.70 ± 6.30% vs. 161.60 ± 5.50%, n = 14) groups (Fig 2B).

**Fig 3:**
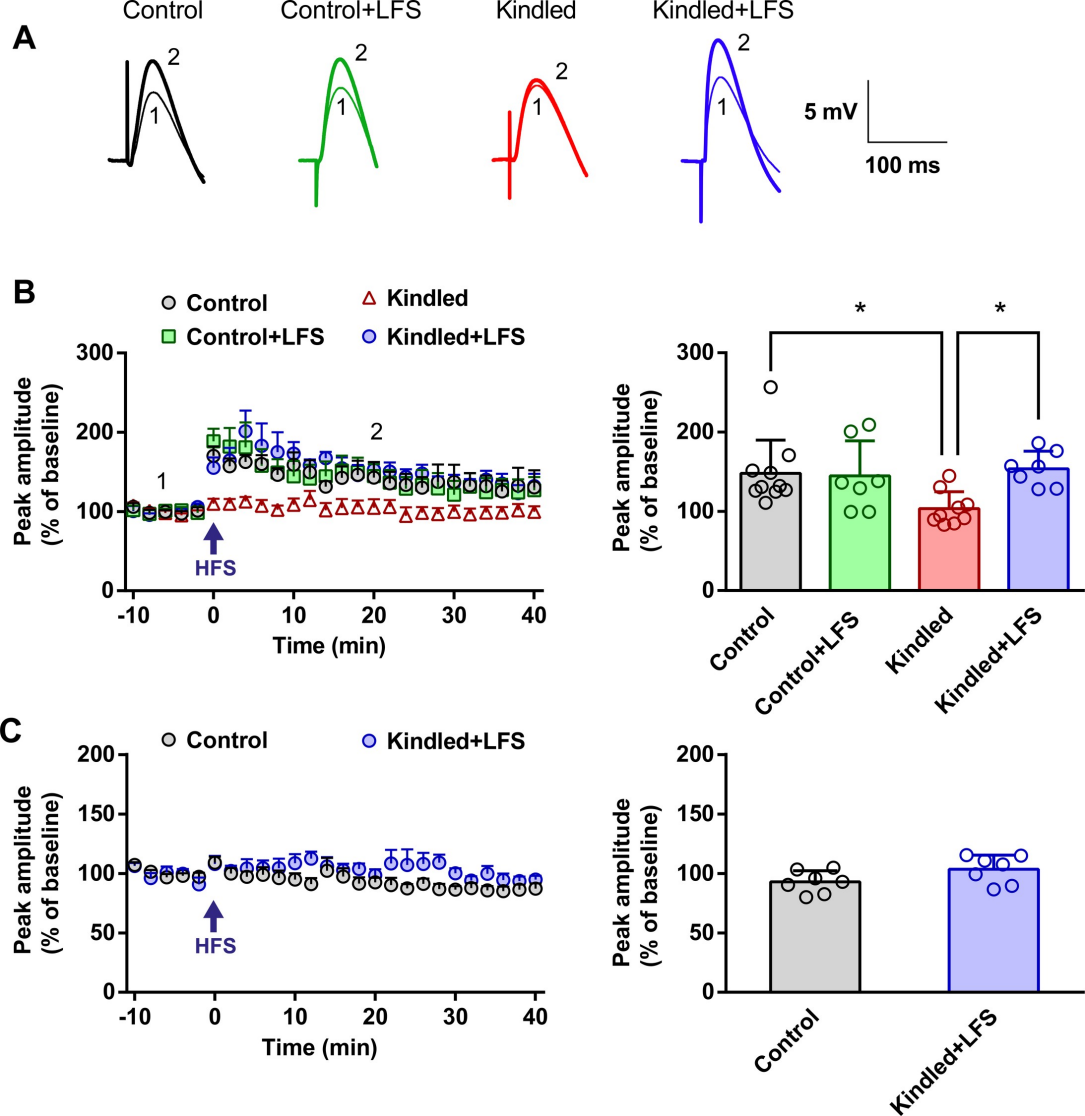
Improvement of iLTP induction in hippocampal slices of kindled animals, following LFS application. (A) IPSPs sample recordings in different experimental groups, before (1) and after (2) HFS application. The numerals near the traces correspond to the times indicated by the same numerals in the graphs. (B) Left: Time course of the IPSP amplitude alteration (% of the baseline) within 40 min after HFS delivery. In the kindled group, iLTP was attenuated significantly compared to the control group. This impairment in iLTP induction was restored following application of LFS in kindled animals. Delivery of LFS alone in control animals did not affect the magnitude of iLTP induction. Right: The bar graph shows the quantitative analysis of iLTP. Control: n = 11, Control+LFS: n = 7, Kindled: n = 9, Kindled+LFS: n = 7. (C) Investigating the role of Ca2+ in iLTP induction in the control and kindled+LFS groups. Left: Time course and Right: bar graph represent that HFS could not induce iLTP in both of control and kindled+LFS groups in the presence of BAPTA (a Ca2+ chelator). Control: n = 7, Kindled+LFS: n = 7. Data are shown as mean±SEM. * p<0.05 compared to control group.

In the next experiment, the impaired potentiation of inhibitory synapses in the hippocampal CA1 region of kindled animals was shown. Evoked IPSPs were recorded in current clamp mode for 10 minutes as a baseline. Then, HFS was applied, and IPSPs were recorded for 40 minutes. In the control group, applying HFS resulted in a significant increase in the IPSP amplitude (147.90 ± 13.22% of baseline, n = 11), while the same pattern of HFS could not induce any changes in the kindled group (103.60 ± 7.11% of baseline, n = 9) (P<0.05). LFS application in kindled animals restored the ability of iLTP induction, so that there was a significant difference between the magnitude of iLTP in this group (153.80 ± 8.40% of baseline, n = 7) and the kindled group (P<0.05). There was no significant difference between the kindled+LFS and control groups. Delivery of LFS alone in the control group (control+LFS) had no significant effect on iLTP induction (144.60 ± 16.75% of baseline, n = 7) compared to the control group (Fig 3B).

To compare the observed iLTP between the kindled+LFS and control group, in the next experiment, we examined the dependency of iLTP to intracellular Ca^2+^. When BAPTA 10 mM (a Ca^2+^chelator) was added to the internal solution, LTP was not induced in the kindled+LFS (103.40 ± 4.47% of baseline, n = 7) or in the control (92.82 ± 3.56% of baseline, n = 7) group; revealing that Ca2+ ion is critical for iLTP induction in the kindled+LFS group as is in the control group (Fig 3C).

**Fig 4:**
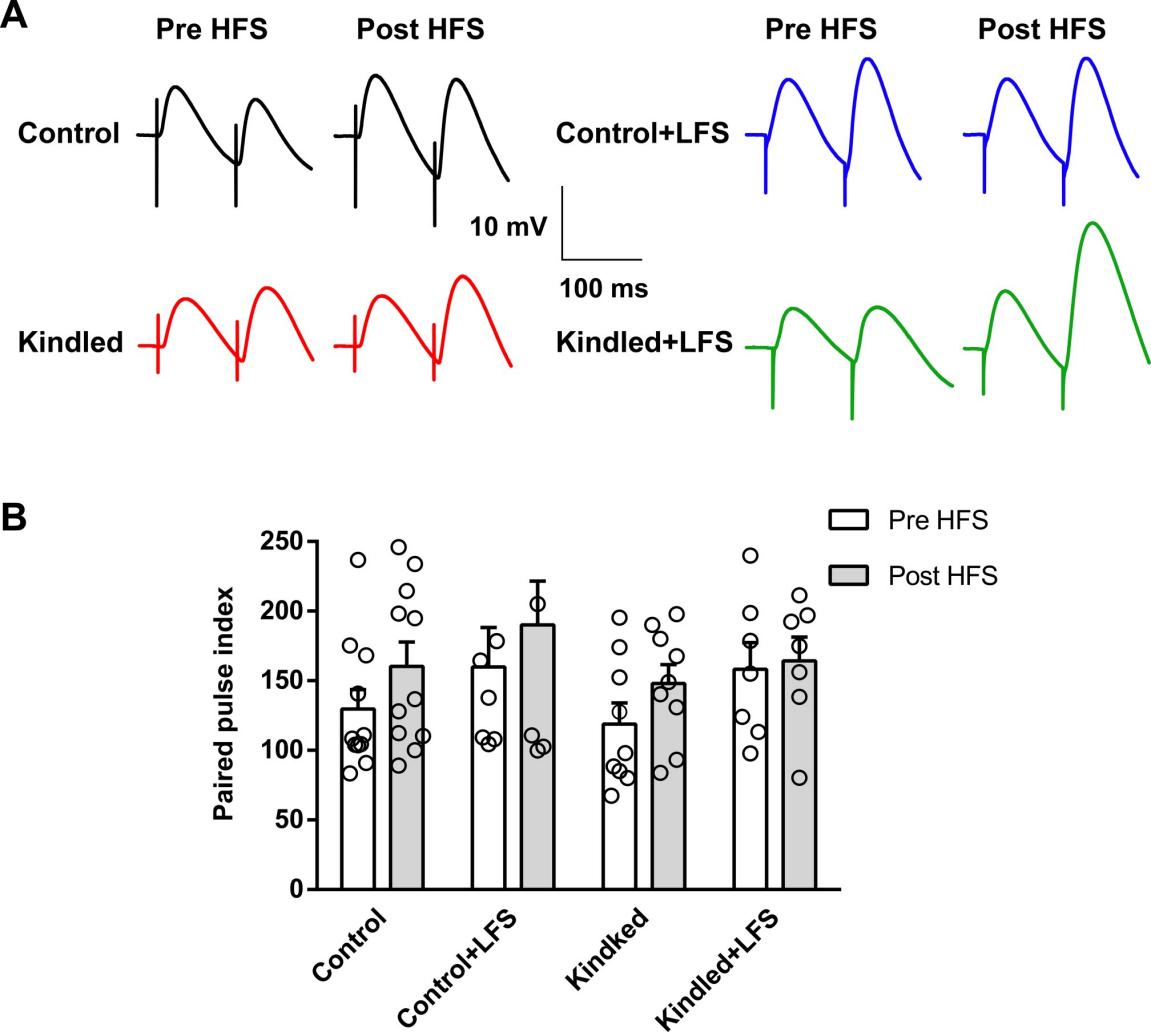
Effect of LFS on PPI of IPSPs before and after HFS application in hippocampal slices of different experimental groups. (A) Sample recording of IPSPs paired pulse in different experimental groups. No significant change in PPI of IPSPs was observed before and after HFS application in the control, control+LFS, kindled or kindled+LFS group. Control: n = 11, Control+LFS: n = 7, Kindled: n = 9, Kindled+LFS: n = 7. Data are shown as mean±SEM.

PPI was also determined to investigate the role of pre- and post-synaptic mechanisms in iLTP induction before and after HFS application. PPI was calculated as the percent ratio of the amplitude of the second IPSP to the first IPSP. A two-way ANOVA revealed no significant difference in the PPI of different experimental groups, both before and after delivery of HFS. Moreover, no significant change in PPI was observed before and after HFS application in controls (129.70±14.00% vs. 160.40±17.40%, n = 11), LFS (159.90±28.40% vs. 190.00±31.60%, n = 7), kindled (118.80±15.25% vs. 148.10±13.50%, n = 9) and kindled+LFS (158.20±19.20% vs. 164.40±16.90%, n = 7) (Fig 4B).

The changes in eEPSCs and eIPSCs may have a role in eLTP and iLTP impairments in the kindled group. Therefore, in the next set of experiments, we investigated their possible role in improving the effect of LFS on eLTP and iLTP events in the kindled+LFS group. For this purpose, the EPSCs and IPSCs were recorded in different experimental groups; evoked EPSCs and IPSCs were recorded at -65 mV every 15 seconds and 6 consecutive responses were averaged for measuring the parameters.

**Fig 5:**
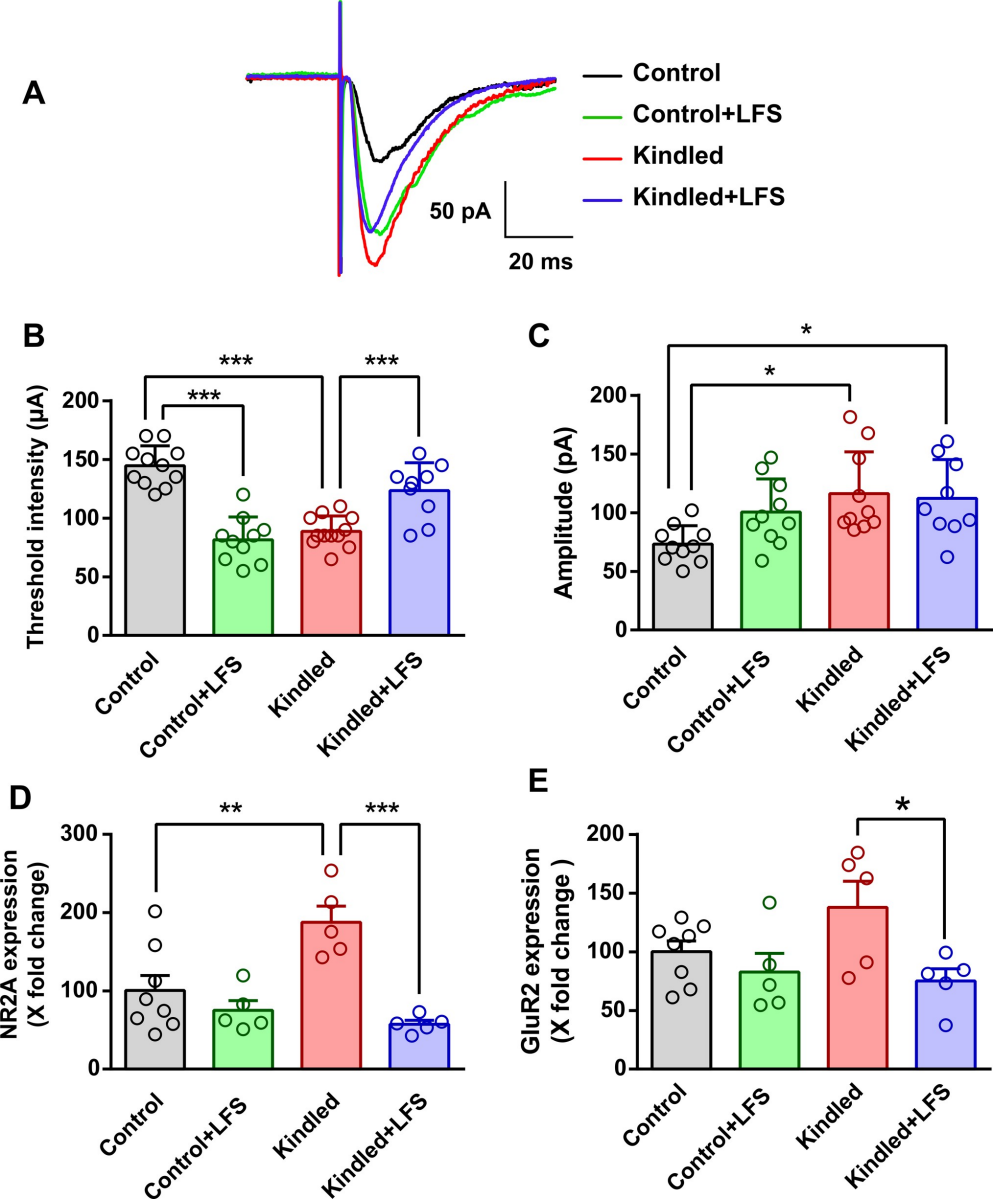
Effect of LFS on currents and gene expression of glutamatergic receptors. (A) Sample recording of evoked EPSCs in different experimental groups. (B) Effect of LFS on the threshold intensity of EPSCs in different experimental groups. In the kindled group, threshold intensity to evoke the EPSC decreased significantly compared to the control group. Application of LFS in kindled animals increased this parameter. Similar to the kindled group, application of LFS alone in control animals decreased the threshold intensity as compared to the control group. Control: n = 10, Control+LFS: n = 10, Kindled: n = 10, Kindled+LFS: n = 9. (C) Effect of LFS on the amplitude of EPSCs in different experimental groups. Amplitude of EPSCs in kindled animals was increased compared to the control group. Application of LFS in kindled animals had no effect on threshold intensity in the kindled+LFS group. Furthermore, delivery of LFS in control animals had no significant effect on EPSC amplitude compared to the control group. Control: n = 10, Control+LFS: n = 10, Kindled: n = 10, Kindled+LFS: n = 9. (D) Effect of LFS on the NR2A subunit of NMDA receptor gene expression in different experimental groups. NR2A gene expression was increased in the kindled group. Application of LFS in kindled animals significantly decreased the expression of this gene in the kindled+LFS group compared to the kindled group, while there was no difference between the kindled+LFS and control groups. In addition, application of LFS alone had no significant effect on NR2A gene expression compare to control group. Control: n = 8, Control+LFS: n = 5, Kindled: n = 5, Kindled+LFS: n = 5. (E) Effect of LFS on the GLUR2 subunit of AMPA receptor gene expression in different experimental groups. Results of this study showed a non-significant increase in GluR2 gene expression in the kindled group compared to the control group. LFS delivery in kindled animals significantly decreased the expression of this gene compared to the kindled group. Control: n = 8, Control+LFS: n = 5, Kindled: n = 5, Kindled+LFS: n = 5. Application of LFS in control animals did not change GluR2 gene expression compared to the control group. Data are shown as mean±SEM. * p<0.05, ** p<0.01 and *** p<0.001 compared to the control group.

A two-way ANOVA revealed that in the kindled group, the threshold intensity to evoke the EPSC (88.80 ± 13.00 μA, n = 10) decreased significantly compared to the control group (144.50 ± 17.10 μA, n = 10) (P<0.001). Application of LFS in kindled animals (kindled+LFS group) increased this parameter (123.30 ± 23.90 μA, n = 9), so that there was no significant difference between the kindled+LFS and control groups. Similar to the kindled group, application of LFS alone in control animals decreased the threshold intensity (81.50 ± 19.40 μA, n = 10) compared to the control group (P<0.001) (Fig 5B). In addition, the amplitude of EPSCs in kindled animals (116.20 ± 35.60 pA, n = 10) increased compared to control group (73.20±15.70 pA, n = 10) (P<0.05). Application of LFS in kindled animals had no effect on this parameter in the kindled+LFS group (122.20 ± 33.10 pA, n = 9). Furthermore, delivery of LFS in the control+LFS group had no significant effect on EPSC amplitude compared to the control group (100.60±28.20 pA, n = 10) (Fig 5C).

**Fig 6:**
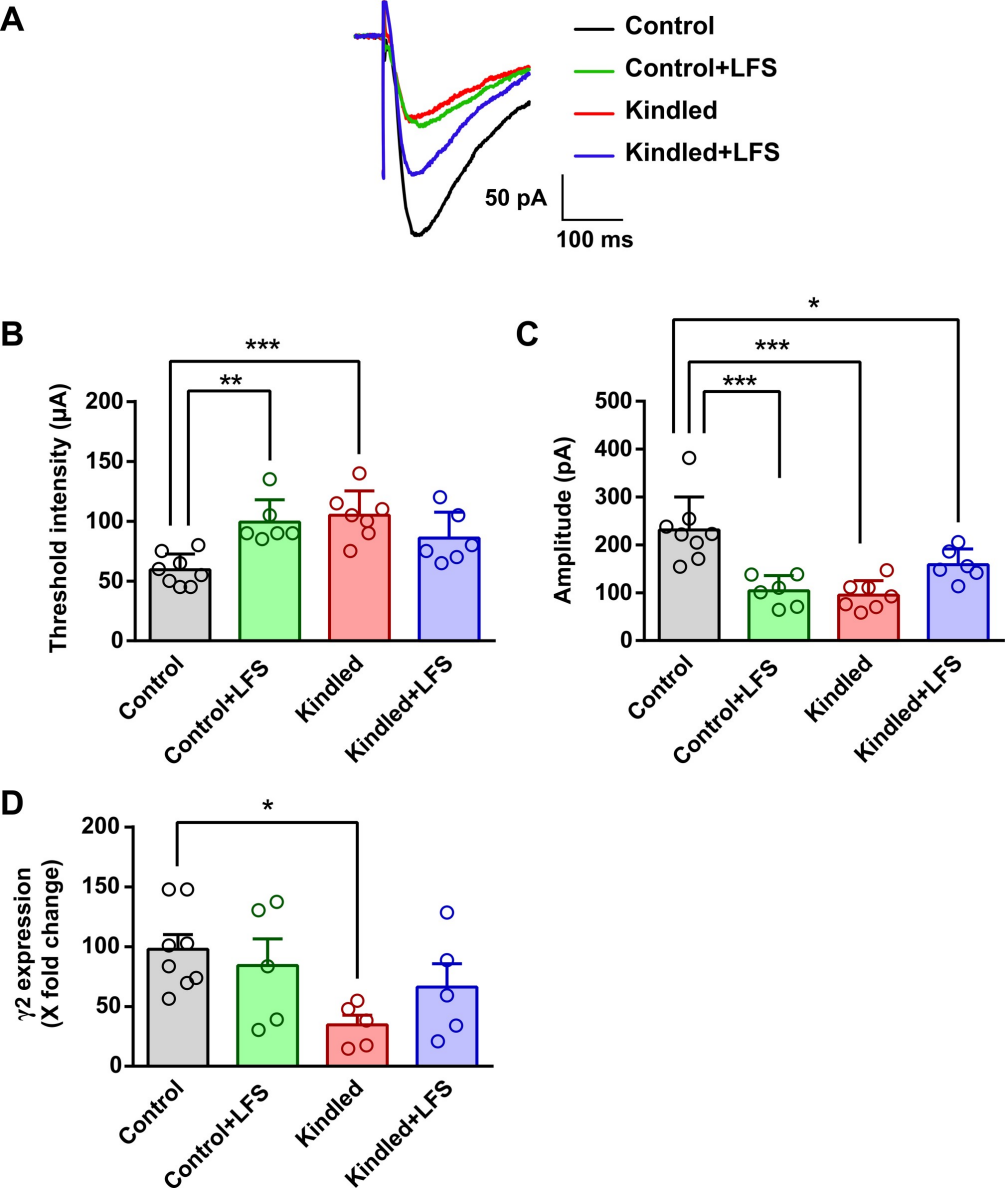
Effect of LFS on currents and gene expression of GABAergic receptors. (A) Sample recording of evoked IPSCs in different experimental groups. (B) Effect of LFS on the threshold intensity of EPSCs in different experimental groups. There was a significant increase in threshold intensity of IPSCs in the kindled group compared to the control group. Application of LFS in kindled animals reduced the IPSC threshold and there was no significant difference in threshold intensity between the kindled+LFS and control group. Threshold intensity in LFS group significantly increased compared to the control group. Control: n = 8, Control+LFS: n = 6, Kindled: n = 7, Kindled+LFS: n = 6. (C) Effect of LFS on the amplitude of IPSCs in different experimental groups. The amplitude of IPSCs was significantly decreased in the kindled and LFS groups compared to the control group. LFS application in kindled animals had no effect on this parameter and there was a significant difference between the kindled+LFS and control groups. Control: n = 8, Control+LFS: n = 6, Kindled: n = 7, Kindled+LFS: n = 6. (D) Effect of LFS on the γ2 subunit of GABAA receptor gene expression in different experimental groups. γ2 gene expression was significantly decreased in the kindled group compared to the control group. Application of LFS in kindled animals restored this impairment and there was no significant difference between the kindled+LFS and control groups in expressing γ_2_ gene. In addition, delivery of LFS in control animals had no effect on expression of γ_2_ gene compared to the control group. Control: n = 8, Control+LFS: n = 5, Kindled: n = 5, Kindled+LFS: n = 5. Data are shown as mean±SEM. * p<0.05, ** p<0.01 and *** p<0.001 compared to the control group.

Unlike EPSCs, there was a significant increase in threshold intensity of IPSCs in the kindled group (105.00 ± 20.41μA, n = 7) compared to the control group (59.40 ± 13.20 μA, n = 8). The application of LFS in kindled animals reduced the IPSC threshold, and there was no significant difference in this parameter between the kindled+LFS (85.80 ± 21.70μA, n = 6) and control groups. Threshold intensity in the LFS group (81.50 ± 19.44 μA, n = 6) was significantly higher compared to the control group (P<0.01) (Fig 6B). The amplitude of IPSCs was significantly lower in the kindled (95.20 ± 30.30 pA, n = 7) and LFS (103.90 ± 32.00 pA, n = 6) groups as compared to the control group (231.00 ± 69.30 pA, n = 8) (P<0.001). LFS application in kindled animals had no effect on this parameter, and there was a significant difference between the kindled+LFS (158.4 ± 33.00 pA, n = 6) and control groups (P<0.05) (Fig 6C).

In the next step, gene expression of NR2A and NR2B subunits of NMDA receptor, GluR2 subunit of AMPA receptor, and γ2 subunit of GABAA receptor were measured. A two-way ANOVA revealed that NR2A gene expression was significantly greater in the kindled group (188.00±20.0% of control; P<0.01, n = 5). Applying LFS in kindled animals (kindled+LFS group) significantly decreased the expression of NR2A gene (57.50±5%, n = 5) as compared to the kindled group (P<0.001), while there was no significant difference between the kindled+LFS and control groups. In addition, application of LFS alone (control+LFS) had no significant effect on NR2A gene expression (75.10 ± 27.60%, n = 5) compared to the control group (Fig 5D).

The results of this study showed a non-significant increase in GluR2 gene expression in the kindled group (138.00 ± 22.00%, n = 5) compared to the control group (100.00 ± 9.10%, n = 8). LFS delivery in kindled animals (kindled+LFS group) significantly decreased the expression of GluR2 gene compared to the kindled group (P<0.05), however no significant change was observed in the kindled+LFS group (75.00±10.00%, n = 5) compared to the control group. Also, applying LFS in control animals (82.70±16.00%, n = 5) did not change GluR2 gene expression compared to the control group (Fig 5E).

Moreover, our results showed significantly lower γ2 gene expression in the kindled group (34.61 ± 17.92%, n = 5) compared to the control group (97.83 ± 34.46%, n = 8) (P<0.05). Application of LFS in kindled animals prevented this impairment, and there was no significant difference between the kindled+LFS (66.21 ± 43.38, n = 5) and control groups in γ2 expression. In addition, delivery of LFS in control animals (84.17 ± 49.72%, n = 5) had no significant effect on γ2 gene expression compared to the control group (Fig 6D).

## Discussion

Our findings indicated that applying LFS in the CA1 region of the dorsal hippocampus restored LTP induction in both excitatory and inhibitory synapses in hippocampal slices of kindled rats. In addition, administration of LFS in kindled animals reduced the changes in EPSCs, IPSCs, and gene expression of NR_2_A subunit of NMDA receptor, GluR_2_ subunit of AMPA receptors and γ_2_ subunit of GABA_A_ receptors in the hippocampus.

Electrophysiological studies of the rodent hippocampus showed that repeated seizure activity has a profound deleterious effect on synaptic plasticity [25, 26]. Apparently seizure activity gradually leads to a random and widespread LTP induction, which in turn reduces the hippocampal for inducing LTP. [26]. This impairment reduces the hippocampal synapses ability to express a new plasticity [13].

In our previous studies, LFS showed an anticonvulsant effect in kindled animals [11, 27, 28] and improved the LTP induction in freely-moving, kindled animals [11, 12]. Probably, LFS can return synapses’ ability to regenerate new LTP through depotentiation mechanisms [29]. Most available information about synaptic plasticity comes from extracellular experiments, so we cannot determine whether the impairment of synaptic plasticity is in excitatory or inhibitory synapses. Therefore, in this study we used patch-clamp recordings to separately investigate excitatory and inhibitory synapses.

In the present study, we checked the effect of either “kindling protocol” or “kindling protocol+LFS” on HFS-induced LTP. LTP induction in Schaffer collaterals to CA1 synapses depends on many mechanisms, including changes in neurotransmission, gene expression, enzymes activities, intracellular signaling and so on. Therefore, applying kindling stimulations or LFS may affect LTP by changing similar or different factors. On the other hand, we previously showed that the LFS pattern used here had anticonvulsant effect in kindled animals. Although the exact mechanism of the LFS anticonvulsant effect has not been completely determined, this phenomenon is accompanied by returning the glutamatergic and GABAergic transmission to normal. Restoring these neurotransmissions may be either a direct mechanism of LFS action or a compensation process. On the whole, direct or indirect restoration of glutamatergic and GABAergic transmission to normal can be considered an important mechanism in LTP induction in the kindled+LFS group of animals.

As we showed in Fig 1, the magnitude of LTP induction in the kindled+LFS group was similar to the control group. In our experiments, all factors that might increase the neural excitability during the kindling procedure could change the amplitude of EPSPs recorded before LTP induction. On the other hand, factors that affected LTP induction and maintenance, including changes in the glutamatergic and GABAergic neurotransmission, could affect the EPSPs after the LTP induction [30, 31]. Therefore, applying LFS altered these states so that LTP induction occurred after HFS administration.

Although eLTP has been examined extensively in the hippocampi of kindled animals [1, 13], knowledge about iLTP in these animals is still limited [32, 33]. Since excitability of CA1 pyramidal neurons is strictly controlled by hippocampal GABAergic interneurons, plasticity of inhibitory synaptic transmission may have a major impact on hippocampal excitability and functionality [34]. In addition, the complementary interactions between the excitatory and inhibitory neurotransmitters (i.e., glutamate and GABA) could form the molecular basis of synaptic plasticity and cognitive performance [35, 36].

Results of the present study showed that the ability of EPSPs potentiation in excitatory synapses was reduced in the kindled group, and application of LFS in kindled animals restored this impairment. In this regard, our previous in vivo experiment showed that LFS can prevent kindling-induced synaptic potentiation in perforant path-granular cells synapses [12]. To investigate the features of induced eLTP in the kindled+LFS group, the role of NMDA receptors in induction and maintenance of eLTP was examined both in the control and kindled+LFS groups. Results of the current study showed that in the kindled+LFS group similar to the control group, NMDA receptors had essential role in eLTP induction, because in the presence of AP5, HFS did not induce eLTP. These receptors had less involvement in the maintenance of eLTP. AP5 administered immediately after applying HFS reduced, but did not abolish, maintenance of eLTP. These results are in line with results of other studies that revealed the important role of NMDA receptors in eLTP induction in excitatory synapses at the CA1 hippocampus region [37].

Of course, it must be noted that in the present study, whole cell recordings were run at room temperature. The temperature can affect activities of neurons in brain slices, and thereby may change several physiological mechanisms involved in synaptic transmission and plasticity. This must be considered a limitation of the present study’s results.

PPI analysis for EPSPs before HFS delivery showed that there was no significant difference in glutamate release between various experimental groups during the basal recording. In addition, there was no significant difference between PPI before and after LTP induction in different experimental groups. This implies that post-synaptic mechanisms may be involved in LTP induction in all groups, and it is likely that LFS through postsynaptic factors could increase the ability of synaptic potentiation in excitatory synapses of kindled rats.

In this regard, our data from kindled animals showed an increased expression of GluR_2_ and NR_2_A genes, which are components of post-synaptic receptors. However, applying LFS significantly prevented the changes in gene expression, which resulted in a significant difference between the kindled and kindled+LFS groups. Moreover, LFS administration significantly inhibited the decrement in the threshold intensity of glutamatergic EPSCs in kindled animals. On the whole, these results are in line with the obtained results of paired-pulse experiments and PPI data, which suggest a probable post-synaptic change in experimental groups.

In the present study, the EPSCs threshold decreased, and the gene expression of NR2A and GluR2 subunits increased in the kindled animals. These changes are consistent with the previous reports that the higher affinities of NMDA and AMPA receptors are likely a reflection of long-term alterations of these receptors’ function in neurons from patients and animals with epilepsy [38–40]. In addition, kindling predominantly increases the mean open time and removes the Mg^2+^ blockade of NMDA receptors [39]. Moreover, up-regulation of NMDA and AMPA receptor-channel complex may be the molecular mechanism that maintains the long-lasting hyperexcitability of hippocampal neurons in kindled animals [40, 41].

Observed changes in NR2A and GluR2 gene expression in the present study are also in line with some previous reports. An enhanced NR2A-containing NMDA receptor expression has been shown in animals and humans with epilepsy [42–46]. In consistent with our results (the decrease in the ability of eLTP induction in kindled animals aligned with the increase in NR2A subunit gene expression), it has been reported that overexpression of NR2A attenuates eLTP induction [47].

It has been suggested that LFS decreases the expression of NR_2_A and GluR_2_ genes back to their normal level through mechanisms involved in depotentiation [29]. NMDA receptors as postsynaptic factors have important role in depotentiation [48]. Therefore, it seems they are involved in the observed effect of LFS in kindled animals. Apparently, following an LFS application, Ca^2+^ enters through NMDA receptors and provides essential Ca^2+^ signaling for inducing depotentiation. Protein phosphatases, especially protein phosphatase1 (PP1) has an important role in depotentiation and Ca^2+^ entry through NMDA receptors PP1, thereby facilitates depotentiation [48, 49]. Some studies have suggested a role for calcineurin and adenosine in PP1 activation [49–51]. Our preliminary experiments revealed a role for calcineurin in LFS action, but supplementary experiments are required [19].

Similar to excitatory neurotransmission, changes in GABA receptor composition, expression, cellular distribution, and function have profound consequences for neural excitability, and they are associated with the etiology of several neurological and mental diseases, including epilepsy [52, 53]. There is much evidence showing the crucial role of GABAergic transmission in generation of epileptic discharges [52, 53]. However, there are different reports about GABAergic system activity after seizure termination. [54, 55]. Evans et al. have reported a marked reduction in GABA_A_ currents in cultured neurons from animals that have experienced three seizures. They also proposed that the observed reduction is likely related to a reduction of the γ2 subunit of GABA_A_ receptor [56]. In addition, Gonzalez et al. reported that number of GABA_A_ receptors (GABA_A_Rs) increased at the plasma membrane in animals that were seizure free within the previous 24 hours. GABA_A_Rs upregulation could compensate for the inhibitory neurotransmission deficit, thereby facilitating a decent inhibitory function. In contrast, although animals with frequent or recent seizures expressed a normal level of GABA_A_Rs at the plasma membrane in comparison to the control group, their tonic inhibitory function was decreased. This phenomenon is possibly due to a reduction in GABA_A_R stability at the plasma membrane of animals with frequent or recent seizures. [57].

A common mechanism observed in temporal lobe epilepsy is depolarizing GABA. This is mainly caused by an alteration in the intracellular chloride concentration stimulations [58]. However, this change in the intracellular ion concentration is completely masked when performing whole-cell, patch clamp recordings. In fact, in our experiments, the best scenario was to mimic the real neuronal conditions (e.g., by using sharp-intracellular recording or perforated patch clamp recording). To create this in our experiments, the exact intracellular concentrations of different ions needed to be measured 24 h after the last kindling stimulation. This was a big limitation in our experimental design. However, it must be considered that we used kindling as a laboratory model of seizures, not epilepsy. Although there are many reports showing the increase in GABAergic neurotransmission following kindled seizures (especially in the dentate gyrus), in the kindling model of seizures, it has been also reported that the GABAergic inhibition decreases during hyperexcitation induced by kindling stimulations [58] and a phenomenon named “failure of inhibition” occurs following kindling [59]. In addition, it has been reported that electrical kindling produces decreased GABAergic inhibition in the hippocampus accompanied by alterations in GABA receptor subunits [60–62]. Interestingly, although in the present study, IPSCs were reduced in the CA1 area when measured 24 h after the last kindled seizures, our recent data showed that IPSCs increased 48 h following the last kindling stimulation (unpublished data). Therefore, it seems that depolarizing GABA in the hippocampal CA1 region may be considered at longer durations, post-kindled seizures.

Our obtained results showed that IPSCs’ threshold intensity increased, and its amplitude decreased 24 hours post kindling. Applying LFS in kindled animals returned the threshold intensity back to its normal value. One possible reason for reduction in GABA currents following kindling could be the decreased expression of GABA receptors in synaptic terminals [55, 63, 64]. Moreover, it has been suggested that the reduction in GABAergic post-synaptic currents is due to the activation of NMDA receptors following epileptic seizures and the elevation of intracellular Ca^2+^ [65]. Our RT-qPCR result showed a significant reduction in the expression of γ_2_ subunit gene in kindled animals, and applying LFS increased γ_2_ expression towards its normal level, therefore there was no significant difference between the kindled+LFS and control groups in γ_2_ gene expression.

In addition to the main role of eLTP in the brain function, plasticity in inhibitory synapses has an important effect on the function of neuronal circuits by altering the excitatory/inhibitory balance [4]. We showed that similar to eLTP, generation of iLTP was also impaired in kindled animals, and applying LFS returned the ability of iLTP induction to the inhibitory circuits in the CA1 region, and the magnitude of iLTP in the kindled+LFS group was same as the control group.

It has been shown that induction of iLTP in the hippocampal CA1 area needs postsynaptic activation of GABA_B_ and mGluR1 receptors [3, 66]. In the current study we used QX314 in the internal solution to prevent the spike generation. However, this chemical is also a GABA_B_ receptor blocker [67]. Therefore, we did not consider a significant role for GABA_B_ receptors in iLTP induction, and the induced iLTP was mainly a result of mGluR1 receptors activation in postsynaptic neurons. In addition, up-regulation of these receptors has been reported 24 hours after the last kindling stimulation in kindled animals [68, 69] (i.e., the time of iLTP recording in our experiments).

Activation of mGluR1 elevates the intracellular Ca^2+^ concentration. Increasing intracellular Ca^2+^ stimulates release of a retrograde messenger, probably glutamate, which can increase GABA release from presynaptic terminals, and finally leads to iLTP in the postsynaptic cell [3, 66]. Therefore, to check whether the role of intracellular Ca^2+^ in iLTP induction in the kindled+LFS group was similar to its role in the control group, BAPTA (10mM) as a Ca^2+^ chelator was added to the internal solution. Results of this experiment showed that iLTP was not induced in the kindled+LFS group or in the control group. Therefore, it may be concluded that intracellular Ca^2+^ has the same critical role for induction of iLTP in both groups.

As we have shown, there was no significant difference in PPI of IPSPs before HFS delivery among various experimental groups, representing no significant difference in GABA release in the basal situation. Therefore, the observed reduction of IPSCs in kindled animals was mainly related to the decrease in the gene expression of γ_2_ receptors in post-synaptic neurons. On the other hand, it could be implied that the mild improvements by LFS on IPSCs (i.e., preventing the kindling-induced increase in the threshold of GABA_A_ receptors) were also due to post-synaptic effects of LFS on GABA_A_ subunits gene expression.

All of the mentioned LFS effects on excitatory and inhibitory synaptic transmissions occurred at the same time that we had observed the anticonvulsant action of LFS in our previous study [27]. Therefore, it may be suggested that the decrease in glutamatergic transmission and, at the same time, the increase in GABAergic activity are among the most important mechanisms involved in the anticonvulsant action of LFS at 24 h post seizures. In fact, these changes may lead to the decrement of excitation/inhibition ratio in kindled animals.

In the current study, PPI was also measured before and after HFS application. There was no significant difference between paired pulse facilitation before and after iLTP induction in different experimental groups. This implies that post-synaptic mechanisms may be involved in iLTP induction in all groups. More experiments are required to determine the exact mechanisms involved how iLTP is improved by LFS.

### Conclusion

Taken together, results of the present study showed that application of LFS in fully kindled animals improved the ability of LTP induction in both excitatory and inhibitory synapses and reduced the kindling-induced changes in glutamatergic and GABAergic synaptic transmission. In addition, LFS delivery in kindled animals reduced NR_2_A and GluR_2_ gene expression and increased γ_2_ gene expression back to normal levels. This effect of LFS can be considered a mechanism for its anticonvulsant action and its consequent improvements on cognitive function in kindled animals.

## Abbreviations

AD: Afterdischarge
ADD: Afterdischarge duration
LFS: Low-frequency stimulation
